# Autism Spectrum Social Stories In Schools Trial 2 (ASSSIST2): study protocol for a randomised controlled trial analysing clinical and cost-effectiveness of Social Stories™ in primary schools

**DOI:** 10.1186/s40359-020-00427-z

**Published:** 2020-06-12

**Authors:** B. Wright, C. Teige, J. Watson, R. Hodkinson, D. Marshall, D. Varley, V. Allgar, L. Mandefield, S. Parrott, E. Kingsley, R. Hargate, N. Mitchell, S. Ali, D. McMillan, H. Wang, C. Hewitt

**Affiliations:** 1 Child Oriented Mental Health Intervention Centre, Leeds and York Partnership NHS Foundation Trust, York, UK; 2 Hull York Medical School, University of York, York, UK; 3 COMIC, IT Centre, Innovation Way, Heslington, York, YO10 5NP UK; 4 York Trials Unit, Department of Health Sciences, University of York, York, UK; 5 Centre for Reviews and Dissemination, University of York, York, UK; 6 Department of Health Sciences, University of York, York, UK; 7 Department of Epidemiology and Biostatistics, Schulich School of Medicine, Western University, London, Ontario Canada

**Keywords:** Social stories, Autism Spectrum conditions, School based interventions, Child mental health, Education

## Abstract

**Background:**

Interventions designed to support children with a diagnosis of Autism Spectrum Conditions (ASC) can be time consuming, needing involvement of outside experts. Social Stories™ are a highly personalised intervention aiming to give children with ASC social information or describing an otherwise difficult situation or skill. This can be delivered daily by staff in education settings. Studies examining Social Story™ use have yielded mostly positive results but have largely been single case studies with a lack of randomised controlled trials (RCTs). Despite this numerous schools are utilising Social Stories™, and a fully powered RCT is timely.

**Methods:**

A multi-site pragmatic cluster RCT comparing care as usual with Social Stories™ and care as usual. This study will recruit 278 participants (aged 4–11) with a clinical diagnosis of ASC, currently attending primary school in the North of England. Approximately 278 school based staff will be recruited to provide school based information about participating children with approximately 140 recruited to deliver the intervention. The study will be cluster randomised by school. Potential participants will be screened for eligibility prior to giving informed consent. Follow up data will be collected at 6 weeks and 6 months post randomisation and will assess changes in participants’ social responsiveness, goal based outcomes, social and emotional health. The primary outcome measure is the Social Responsiveness Scale Second Edition (SRS-2) completed by school based staff at 6 months. Approvals have been obtained from the University of York’s Research Governance Committee, Research Ethics Committee and the Health Research Authority. Study results will be submitted for publication in peer-reviewed journals and disseminated to participating families, educational staff, local authority representatives, community groups and Patient and Participant Involvement representatives. Suggestions will be made to NICE about treatment evidence dependent on findings.

**Discussion:**

This study addresses a much used but currently under researched intervention and results will inform school based support for primary school children with a diagnosis of ASC.

**Trial registration:**

The trial is registered on the ISRCTN registry (registration number: ISRCTN11634810). The trial was retrospectively registered on 23rd April 2019.

## Background

Autism Spectrum Disorders, often termed Autism Spectrum Conditions (ASC) [1] affects at least 1% of children in the UK [2]. ASC is a lifelong neurodevelopmental condition that can impact upon a range of child and adult outcomes [3, 4]. Children with ASC have a range of difficulties with social communication, sensory processing differences, and may have repetitive behaviours [5] and/or preoccupations [6]. They experience a higher prevalence of mental health problems than typically developing children including anxiety and low mood [7]. Many children with ASC struggle to manage social anxiety and feelings of frustration, which can lead to challenging behaviour [8–10]. Research shows that many people with ASC have additional care needs, both in the NHS and social care across childhood and adulthood [11, 12].

Social norms and classroom expectations can be difficult for children with ASC to learn and understand [13] and teachers facing many demands on their time and with limited training opportunities, may struggle to support their education [14].

Carol Gray’s Social Stories™ are short, highly individualised stories that provide children with social information. They help children with ASC more easily understand a situation personal to them such as learning life skills, new experiences, understanding emotions (or people), transitions or change. The child is included in the narrative often with a range of helpful visual information. They are child friendly and do not require extensive involvement from outside experts [15]. Social Stories™ are defined by ten criteria that guide story development [16]. These help to ensure stories remain patient and supportive in tone using relevant, tailored content that is descriptive, meaningful, and safe for the child. The capacity for tailoring these stories is particularly important for helping children with ASC across a range of ages, strengths, difficulties and needs [17, 18]. Previous research examining Social Story™ use in specialist and mainstream education [19, 20] and within the home [18] shows largely positive results [21], and suggests that Social Stories™ may promote calmer classrooms with improved learning and better integration in Special Educational Needs (SEN) [19] and mainstream settings [20]. Children with ASC have shown improvements in social interactions [22–24], decision making [23], self awareness in communication [24] and mealtime skills [25]. Success has also been reported in reducing tantrums [26, 27] and managing frustration [28]. This research also suggests that it is possible to train tier one professionals, for example teachers, to develop and use Social Stories™ tailored to a child [29].

Despite this research, two systematic reviews of their effectiveness [30, 31] indicate large gaps in the literature. The reviews mainly identify single case designs and a lack of randomised controlled trial evidence.

This research is needed now because numerous schools utilise Social Stories™ despite a limited evidence base. This is highly relevant when many providers face limited resources for children with neurodevelopmental problems and mental health problems. Given that specialist practitioner interventions are in short supply, interventions such as Social Stories™ deserve robust evaluation as they can be delivered within schools on a day to day basis. We will be exploring these aspects within our study. This study follows an RCT that assessed the feasibility and acceptability of running a trial examining Social Story™ use in both primary and secondary mainstream schools [32]. This demonstrated a high degree of acceptability with young people, family and schools. This main trial now seeks to assess the clinical and cost-effectiveness of Social Stories™, addressing the lack of fully powered RCTs in this area.

### Study aim

To assess the clinical and cost-effectiveness of Social Stories™ alongside care as usual in primary schools when compared with care as usual alone.

### Study objectives

#### Primary

The primary objective of the study is to establish whether Social Stories™ can improve social responsiveness in children with ASC in primary schools across Yorkshire and the Humber, when compared to children who have received care as usual only.

#### Secondary

The secondary objectives of this trial are:

1. To investigate whether Social Stories™ can reduce challenging behaviour in children with ASC in primary schools.
2. To investigate whether Social Stories™ can improve social and emotional health in children with ASC in primary schools.
3. To assess the cost-effectiveness of Social Stories™.
4. To examine the effects of Social Stories™ delivered in the classroom on general measures of health related quality of life.
5. To examine whether Social Stories™ improve classroom attendance.
6. To assess sustainability of Social Stories™ in an education setting across a 6 month period.
7. To examine any changes in parental stress.
8. To examine any associations between treatment preference and outcomes.
9. To examine how elements of session delivery (e.g. session frequency, length and any associated problems/adverse events) are associated with outcomes.

### Trial design

This trial is a multi-site pragmatic cluster RCT comparing Social Stories™ and care as usual with a control group receiving care as usual alone. Care as usual is defined as the existing support routinely provided for a child with ASC from educational and health services such as specialist autism teacher teams, mental health teams or other associated professionals. The trial includes a 10 month internal pilot, a process evaluation (including qualitative interviews and an examination of treatment fidelity) and an economic evaluation.

## Methods

### Study setting

Participants will be recruited using a variety of methods including identification through schools, or identification through participating NHS trusts using their clinic lists/databases. Intervention delivery will take place in the participant’s school.

### Eligibility criteria

Both the school and the child’s parents/guardians must agree to take part before either may be included. Eligibility to take part will be ascertained using the following criteria.

Inclusion criteria:

- The child is aged 4–11 years.
- The child attends a participating primary school within Yorkshire and the Humber.
- The child has a clinical diagnosis of ASC and daily challenging behaviour.
- Parents/guardians of the child are able to self-complete the English language outcome measures (with assistance if necessary).

Exclusion criteria:

- The school has used Social Stories™ for any pupil in the current or preceding school term.
- The child or interventionist teacher has taken part in the previous Social Stories™ feasibility study (ASSSIST). Schools that have taken part will not be excluded.

### Intervention

Children in the intervention arm of the trial will receive the Social Stories™ intervention in addition to their care as usual.

The Social Stories™ intervention will be delivered by a trained educational professional (the interventionist) who is employed within each school allocated to the intervention arm. The interventionist may vary between the schools (e.g. a teacher, teaching assistant (TA), or Special Educational Needs Coordinator (SENCO)). Social Stories™ used within the trial will be based on a goal developed mainly by an education professional who knows the participating child well but is not the interventionist (the associated teacher). This goal will be developed with support from the study team; and where possible the parent/guardian, interventionist and the child. A Social Story™ is used to provide a child with social information and works well if a child is uncertain or finds a situation distressing, frustrating or incomprehensible.

Interventionists will be trained by the research team or through trained local authority autism specialist staff. Training will include information on the design and implementation of Social Stories™, with materials based on those developed in the preceding feasibility study with the support of Carol Gray [32] and a Social Stories™ manual produced by the study’s Chief Investigator (CI) and a Clinical Psychologist with expertise in autism [33]. During the training session, interventionists will construct a Social Story™. Parents/guardians will also be invited to attend these sessions. Following training all Social Stories™ will be assessed against a fidelity checklist, to ensure they conform to the ten established criteria [16, 33]. They will then be delivered to children in the intervention arm by the interventionist at least six times over a 4 week period.

Goal based outcome measures will be used and 20 % of children allocated to the intervention arm will be observed by a blinded research assistant to ascertain their progress towards the goal. Consent to these observations will be optional and participants will be randomly selected by the Trials Unit using a computer generated list from those who have opted in.

### Control

Participants allocated to the control arm of the trial will receive care as usual. Schools in the control arm are asked to refrain from delivering any Social Stories™ for the duration of their trial involvement.

If a participant decides to withdraw from the intervention and/or the study, a member of the research team will record details including reasons provided, whether withdrawal is from the whole study or an individual element, and whether they wish to continue providing data for analysis.

### Outcomes (primary and secondary)

Baseline and follow-up measures will be collected during visits to schools, participants’ homes or at locations convenient to participants or via post where face to face data collection is not possible. The primary outcome is the Social Responsiveness Scale, Second Edition (SRS-2) [34] completed at 6 months post-randomisation by the associated teacher. The SRS-2 measures social interaction skills and ASC symptomatology.

**Fig. 1:**
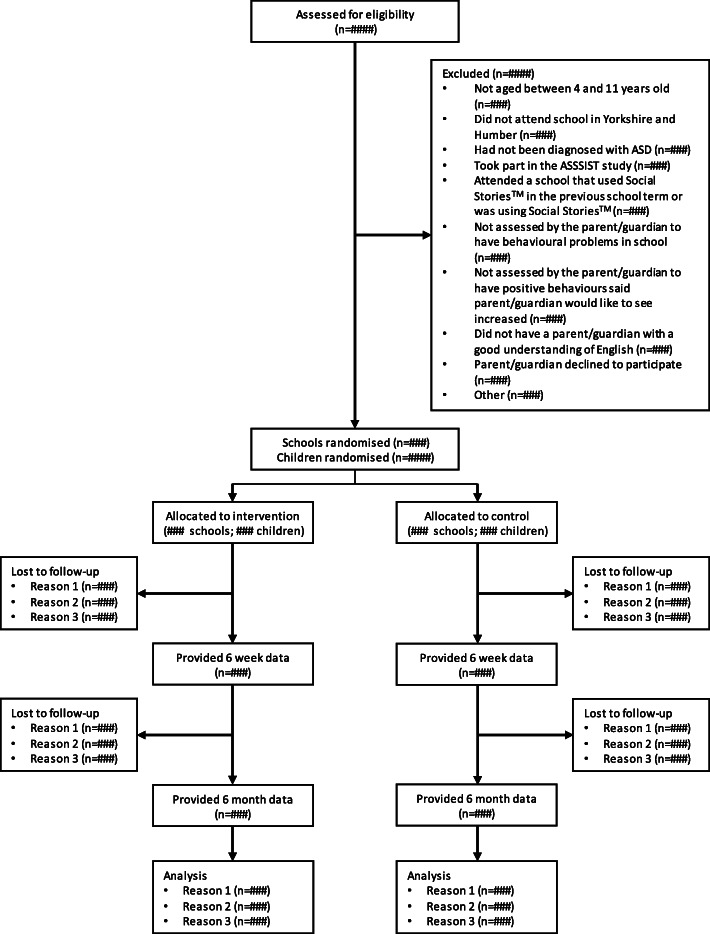
Study Flow Chart

All secondary outcomes are listed below (grouped by respondent) and will be collected at baseline, 6 weeks and 6 months post-randomisation unless specified. The trial flow chart is seen in Fig. 1.

Parent questionnaires:

1. Social Responsiveness Scale, Second Edition (SRS-2) [34].
2. Demographic information pertaining to the child and the parent – *baseline only.*
3. Parenting Stress Index short form [35].
4. The EQ-5D-Y (proxy) [36].
5. Revised Children Anxiety and Depression Scale (RCADS) short form [37].
6. Bespoke resource use questionnaire, capturing healthcare and non-health resource use of participants and parents/carers– *baseline and 6 months only.*
7. Bespoke treatment preference questionnaire – *baseline only.*

Associated teacher/TA questionnaires:

1. Social Responsiveness Scale, Second Edition (SRS-2) [34].
2. A goal-based outcome measure (adapted from the Child Outcomes Research Consortium) [38].
3. Bespoke resource use questionnaire – *baseline and 6 months only.*
4. Bespoke treatment preference questionnaire – *baseline only.*
5. Bespoke resource use questionnaire determining current school care/education plan interventions – *baseline and 6 months only.*

Interventionist Teacher/ TA Questionnaires:

1. Bespoke Social Story™ session log. – *used after each Social Story™ session.*
2. A bespoke sustainability questionnaire – *6 weeks and 6 months only.*

Process Evaluation - Researcher Questionnaires:

1. A fidelity checklist (intervention arm only) assessing each Social Story™ - *after the story has been created and at 6 weeks post-randomisation*.
2. A goal-based measure – used when observing randomised children at 6 weeks and 6 months.

### Sample size calculation

The primary outcome is the teacher completed SRS-2 t-score at 6 months. Within the pilot data, outcomes were measured at 6 and 16 weeks. The correlation between baseline and 6 weeks (*r* = 0.67, 95% CI 0.44 to 0.80) was lower than that at 16 weeks (*r* = 0.83, 95% CI 0.68 to 0.91) for the pilot data. To be conservative we have chosen the lower 95% confidence limit for the lowest correlation between baseline and follow-up that we observed within our pilot data (*r* = 0.44). Assuming a difference of 3 points, SD = 7, 5% alpha, 90% power, average cluster size 1.35, ICC = 0.34, correlation = 0.44 and 25% attrition requires a total sample size of 278.

### Recruitment

The study aims to recruit 278 children with ASC from across Yorkshire and the Humber. Recruitment opened in November 2018. In addition, the study will recruit 278 parents/guardians, 278 associated teachers and approximately 140 interventionists who will each complete study questionnaires, and in the case of interventionists, deliver Social Stories™ sessions.

ASSSIST2 will use five methods of participant recruitment outlined in more detail below:

#### Recruitment from schools

Primary schools across Yorkshire and the Humber will be contacted with information about the trial. Where schools agree to participate they will be asked to send information sheets and Expression of Interest (EOI) forms to parents/guardians of eligible children. Parents/guardians who return an EOI form or have consented for school staff to pass on their details will be contacted by a member of the research team to discuss the trial, answer any questions and to arrange collection of informed consent from parents/guardians and where possible assent from the child. We will also gain consent from education professionals taking part in the trial.

#### Recruitment from NHS sites

Participating NHS Trusts who carry out autism spectrum assessments will be asked to identify potentially eligible families through screening of their clinic/database lists. A member of the clinic team (or delegated staff member) will send study information and EOI forms to the child’s parent/guardian. Recruitment will then follow the procedure set out for schools.

#### Recruitment from local community groups

Local community groups within the catchment area, e.g. the Autism Spectrum Conditions - Enhancing Nurture and Development (ASCEND) parent group [39], will be contacted with information about the trial and researchers will distribute study information to interested parents/guardians. On receipt of an EOI, recruitment will follow the procedure outlined for schools.

#### Liaising with local authority professionals

Relevant local authority professionals, e.g. educational specialists in ASC, will be contacted and asked to disseminate study information and EOI forms to the parents/guardians of potentially eligible children. On receipt of an EOI recruitment will follow the procedure outlined for schools.

#### Recruitment from local publicity

Families may hear about the trial from a range of sources and contact the research team. In this instance the team will confirm the child’s eligibility and send out study information. It will be made clear that participation is dependent on the child’s school taking part and recruitment will continue in line with the procedure outlined for schools.

### Randomisation

Randomisation will occur after consent/assent and baseline data has been obtained from all participating families and educational professionals within a school. Remote randomisation for the trial will be carried out by the York Trials Unit (YTU). Schools will be randomised using cluster randomisation to reduce the risk of contamination within schools with multiple participating children. Randomisation will be stratified by school type (SEN school or mainstream school) and by the number of participating children within a school (≤5 or > 5 participating children).

Following randomisation schools will be notified of study allocation via phone call, email and following letter. Participating parents/guardians will receive a confirmatory letter.

### Blinding

Research assistants collecting outcome data and the main trial statistician will be blinded to study allocation until final data analysis. It is not possible for children, parents/guardians or school staff to be blinded to study allocation due to the nature of the intervention. The Data Monitoring and Ethics Committee (DMEC) will have access to un-blinded data throughout the study. Any instances of un-blinding will be recorded using a bespoke form. In such instances a substitute blinded research assistant will collect participant data wherever possible.

### Data collection

Data collection will utilise paper Case Report Forms (CRFs). To maintain participant confidentiality CRFs will be anonymised using participant ID numbers. Data collection will occur at baseline, 6 weeks and 6 months post-randomisation. Wherever possible baseline CRFs will be completed in person and where not possible, participants will be offered the option to return them by post.

### Data management

All information collected during the study will be kept strictly confidential and stored on a secure password protected server located at the University of York, for the purposes of assisting in follow-ups during the study. All paper documents will be stored securely.

CRFs will be initially checked for errors by the research team and any queries raised immediately with participants. CRFs will then be logged on the YTUs bespoke data management system and scanned using Cardiff Teleform. Original datasheets will be securely stored at YTU. All data will be collected and retained in accordance with the Data Protection Act 2018, the General Data Protection Regulation 2018 and YTU standard operating procedures (SOPs). All data will be archived for 10 years following the end of the study and then securely destroyed.

### Statistical analysis

Statistical analyses will be conducted using a validated statistical software package and will be reported in line with the Consolidated Standards of Reporting Trials (CONSORT) 2010 Statement [40]. The primary analysis population will be intention to treat (ITT).

#### Primary and secondary outcomes analysis

The primary analysis will compare teacher reported SRS-2 t-scores measured at 6 months between the randomised groups using a covariance pattern mixed model. Important baseline covariates, baseline SRS-2 t-score, the SEN status of the child’s school (SEN vs. non-SEN), the number of recruited children in the child’s school (> 5 vs. ≤5), time point, treatment group, and a treatment group-by-time point interaction will be included as fixed effects and school will be included as a random effect. SRS-2 t-scores at 6 weeks post randomisation will be incorporated as outcome data. The model will account for the correlation of scores within pupil over time by means of an appropriate covariance structure. Intervention effects in the form of an adjusted mean difference will be presented with an associated 95% CI and *p*-value for both time points (6 weeks and 6 months).

Secondary outcomes will be analysed in a similar manner to the primary analysis, adjusting for the appropriate baseline score being tested. The summary of Adverse Events (AEs) and Serious Adverse Events (SAEs) experienced by participants will be reported by treatment group.

Subgroup analyses will be performed to explore the potential effect of teacher’s preferred randomisation group collected at baseline. The primary analysis model will be refitted with an interaction term between the randomisation group and teacher’s preference. A Complier Average Causal Effect analysis for the primary outcome will be considered to account for compliance with the intervention at the student level.

#### Process evaluation

The process evaluation will include an interventionist questionnaire, capturing how many times interventionists have used Social Stories™; and whether they have trained others. Interviews and fidelity assessment will be carried out. A Social Story™ writing and delivery training DVD will be developed and an FAQ document identifying elements of good practice in the implementation of Social Stories™ as well as any common issues or barriers highlighted by interventionists and/or parents. Both of these outputs will be freely available on the research team’s website.

#### Qualitative analysis

Semi-structured interviews will be undertaken with 10–20 participants to explore challenges and barriers to training and implementation of Social Stories™, and the willingness of school staff to run the intervention independently. These participants will be purposively selected from the cohort of teachers, trainers, interventionists, Associated Teachers, local authority representatives involved in the trial and parents who attended the training. With consent, all interviews will be recorded on encrypted devices, transcribed verbatim and anonymised. A sample of data transcripts will be checked against audio recordings for accuracy. Interview material will be organised according to analytical headings using a constant comparison approach [41]. Qualitative data analysis computer software will be used to structure and explore the interview data. Electronic sound files and transcripts from qualitative interviews will be assigned a unique participant number, known only to relevant members of the research team. Any quotes published will be anonymous.

#### Fidelity analysis

The fidelity evaluation will examine the extent to which the components of the intervention (Social Stories™) were delivered as planned, and the accommodations required by schools to ensure this. Interventionists’ adherence to core components will be assessed via researcher completed fidelity checklists of each Social Story™ after initial writing and at 6 weeks.

#### Economic analysis

The economic analysis will be conducted from an NHS and Personal Social (PSS) perspective, taking the form of a within-trial cost-effectiveness analysis determining the incremental cost per unit of outcome effectiveness measure for Social Stories™ compared with care as usual in children with autism. Health outcomes will be measured in terms of quality-adjusted life-years (QALYs) using the EQ-5D-Y (proxy version) as a health descriptor measure (the preferred instrument in the NICE reference case). The domains of the EQ-5D-Y proxy will then be valued using UK population tariff to provide utility scores at multiple time points. QALYs will be estimated using time weighted averages of the utility scores measured over the study time period.

The cost of the Social Stories™ intervention will be calculated using a bottom-up estimation of the time spent by professionals delivering the intervention, the cost of training and other resources used. Unit costs of health service use will be obtained from the UK national database of reference costs. The cost of social services will be calculated from the Unit Costs of Health and Social Care, produced by the Personal Social Services Research Unit, and the cost of other professional support will be estimated from relevant salary scales and published reports/ literature.

A sensitivity analysis will be conducted including costs of productivity loss.

### Data monitoring

The conduct of the study will be governed by three committees.

- A Trial Steering Committee (TSC).
- An independent Data Monitoring and Ethics Committee (DMEC).
- A Trial Management Group (TMG).

These committees will function in accordance with YTU SOPs. The DMEC and TSC are both independent from the sponsor. The TSC will consist of an independent chair, an independent subject specialist, an independent clinical academic, an independent statistician and a Patient and Public Involvement (PPI) representative. The DMEC will consist of an independent chair, an independent statistician, and another independent member experienced in research with children and families. The TSC and DMEC will meet approximately every 6 months from the start of the trial. The TMG will comprise co-applicants, members of the trial team (including the data manager), PPI representatives, and the trial managers. Co-applicants and trial team members will be invited as required depending on their roles.

### Adverse event reporting

Possible harm as a result of the study will be monitored according to YTU SOPs. Any Adverse Events (AEs) reported by individuals participating in the study will be recorded using a bespoke Adverse Events Recording Form and assessed for seriousness. AEs will be recorded as a Serious Adverse Event (SAE) if it results in death, is life threatening, prolongs or requires hospitalisation, or results in disability or incapacity. Any AEs relating to study participation and all SAEs will be reported to the CI. SAEs related to the study will be reported to the study Sponsor, DMEC and TSC.

The DMEC will review data throughout the trial for safety. If there is evidence of harm due to the intervention or measures used, the DMEC will advise the TSC with a possible recommendation to stop the trial.

### Protocol amendments

Prior to submission for ethical approval, protocol amendments will be approved by the CI, substantial amendments will be approved by the CI, Sponsor and TMG. Amendment history will be tracked by adopting version control and by the use of an amendment log.

### Dissemination

Results will be published in mainstream and specialist science journals. Study findings will be presented at relevant research conferences, symposia and seminars. In addition, the National Autistic Society and members of service user groups such as ASCEND will be consulted in the development of methods for dissemination that will be effective in reaching families of children with ASC. We will also produce a short summary of results that can be distributed to trial participants as well as relevant interest and patient groups. We will publish findings on relevant websites such as the National Autistic Society, university and child mental health websites. We aim to ensure coverage of our findings in the wider media by issuing a press release.

Towards the end of the trial, our PPI representatives will organise a meeting with stakeholders including parents and professionals working with young people who have ASC to discuss the dissemination of the study findings. We will hold a research dissemination event for national and local clinicians and policy makers. Depending on findings, we will make suggestions to NICE about treatment evidence.

## Discussion

This study aims to examine the clinical and cost effectiveness of Social Stories™ intervention when used in education settings with primary school children. This large scale trial builds upon the initial feasibility work conducted [32], and will enhance a growing body of promising literature [15, 18–31] by comparing the outcomes of children receiving care as usual only and children receiving Social Stories™ in addition to care as usual in a fully powered RCT. Results from this study will help to inform school based interventions for children with a diagnosis of ASC.

### Study limitations

Due to the nature of the intervention it is not possible for participants to be blinded to study allocation, however the trial research assistants well as the trial statistician remain blinded to help mitigate any potential impacts. Further this study focusses solely on the use of Social Stories™ in education based settings, dependent on study findings, further randomised research into the use of Social Stories™ in the home may prove beneficial.

## Data Availability

Anonymised participant data from the ASSSIST2 trial will be made available via the sponsor. Those wishing to access this data must apply to the sponsor and show that they have sound scientific reasons for requesting access to the data as well as having acceptable research methods. Other documents that will be made available include the study’s protocol, statistical analysis plan, health economics plan as well as the case report forms used throughout the study. This data will become available following publication.

## Abbreviations

AEs: Adverse Events
ASC: Autism Spectrum Conditions
ASCEND: Autism Spectrum Conditions – Enhancing Nurture and Development
ASSSIST2: Autism Spectrum Social Stories In Schools Trial 2
CACE: Complier Average Causal Effect
CI: Chief Investigator
CONSORT: Consolidated Standards of Reporting Trials
CRFs: Case Report Forms
DMEC: Data Monitoring and Ethics Committee
EOI: Expression of Interest
HRA: Health Research Authority
ISRCTN: International Standardised Randomised Controlled Trial Number
ITT: Intention to treat
NICE: The National Institute for Health and Care Excellence
NIHR: National Institute of Health
PPI: Patient and Public Involvement
PSS: Personal Social
QALYs: Quality Adjusted Life Years
RCADS: Revised Children Anxiety and Depression Scale
RCT(s): Randomised Controlled Trial(s)
SAEs: Serious Adverse Events
SEN: Special Educational Needs
SENCO: Special Educational Needs Coordinator
SOPs: Standard Operating Procedures
SRS-2: Social Responsiveness Scale Second Edition
TA: Teaching Assistant
TMG: Trial Management Group
TSC: Trial Steering Committee
YTU: York Trials Unit

## References

[1] Baron-Cohen S. ASD vs. ASC: Is One Small Letter Important? In: Presented at International Society for Autism Research Conference. Salt Lake City; 2015. https://insar.confex.com/imfar/2015/webprogram/Paper19861.html. Accessed 1 Feb 2020.

[2] Baird G, Simonoff E, Pickles A, Chandler S, Loucas T, Meldrum D (2006). Prevalence of disorders of the autism spectrum in a population cohort of children in South Thames: the Special Needs and Autism Project (SNAP). Lancet.

[3] Russell G, Rodgers LR, Ukoumunne OC, Ford T (2014). Prevalence of parent-reported ASD and ADHD in the UK: findings from the Millennium Cohort Study. J Autism Dev Disord.

[4] Baron-Cohen S, Scott F, Allison C, Willaims J, Bolton P, Matthews FE (2009). Prevalence of autism spectrum condition: UK school-based population study. Br J Psychiatry.

[5] Turner M (1999). Repetitive behaviour in autism: a review of psychological research. J Child Psychol Psychiatry.

[6] American Psychiatric Association. Diagnostic and statistical manual of mental disorders. 5th ed. Washington, DC: APA Press; 2013.

[7] Kim JA, Szatmari P, Bryson SE, Streiner DL, Wilson FJ (2000). The prevalence of anxiety and mood problems among children with autism and Asperger syndrome. Autism.

[8] Koegel LK, Koegel RL, Hurley C, Frea WD (1992). Improving social skills and disruptive behavior in children with autism through self-management. J Appl Behav Anal.

[9] White SW, Kreiser NL, Pugliese C, Scarpa A (2012). Social anxiety mediates the effect of autism spectrum disorder characteristics on hostility in young adults. Autism.

[10] Donno R, Parker G, Gilmour J, Skuse DH (2010). Social communication deficits in disruptive primary-school children. Br J Psychiatry.

[11] National Institute for Health and Care Excellence (2013). The management and support of children and young people on the autism spectrum.

[12] National Audit Office (2009). Annual Report.

[13] Travis L, Sigman M, Ruskin E (2001). Links between social understanding and social behaviour in verbally able children with autism. J Autism Dev Disord.

[14] Simpson R, De Boer-Ott S, Smith-Myles B (2003). Inclusion of learners with Autism Spectrum Disorders in general education settings. Top Lang Disord.

[15] Scattone D, Tingstrom D, Wilczynski S (2006). Increasing appropriate social interactions of children with Autism Spectrum Disorders using Social Stories™. Focus Autism Other Dev Disabl.

[16] Gray C (2010). The new social story book.

[17] National Research Council (2001). Educating Children with Autism.

[18] Ivey M, Heflin L, Alberto P (2004). The use of Social Stories™ to promote independent behaviors in novel events for children with PDD-NOS. Focus Autism Other Dev Disabl.

[19] Crozier S, Tincani M (2007). Effects of Social Stories™ on prosocial behaviour of preschool children with Autism Spectrum Disorders. J Autism Dev Disord.

[20] Delano M, Snell M (2006). The effects of Social Stories™ on the social engagement of children with autism. J Posit Behav Interv.

[21] Chan J, O'Reilly M (2008). A Social Stories™ intervention package for students with autism in inclusive classroom settings. J Appl Behav Anal.

[22] Norris C, Dattilo J (1999). Evaluating effects of a Social Story™ intervention on a young girl with autism. Focus Autism Other Dev Disabl.

[23] Barry L, Burlew S (2004). Using Social Stories™ to teach choice and play skills to children with autism. Focus Autism Other Dev Disabl.

[24] Ozdemir S (2008). The effectiveness of Social Stories™ on decreasing disruptive behaviors of children with autism: Three case studies. J Autism Dev Disord.

[25] Bledsoe R, Myles B, Simpson R (2003). Use of a Social Story™ intervention to improve mealtime skills of an adolescent with Asperger syndrome. Autism.

[26] Lorimer P, Simpson R, Myles B, Ganz JB (2002). The use of Social Stories™ as a preventative behavioral intervention in a home setting with a child with autism. J Posit Behav Interv.

[27] Kuttler S, Myles BS, Carlson JK (1998). The use of Social Stories™ to reduce precursors to tantrum behaviours in a student with autism. Focus Autism Other Dev Disabl.

[28] Adams L, Gouvousis A, VanLue M, Waldron C (2004). Social Story™ intervention: improving communication skills in a child with an Autism Spectrum Disorder. Focus Autism Other Dev Disabl.

[29] Quilty K (2007). Teaching paraprofessionals how to write and implement Social Stories™ for students with Autism Spectrum Disorders. Remedial Spec Educ.

[30] Kokina A, Kern L (2010). Social Story™ interventions for students with Autism Spectrum Disorders: A meta-analysis. J Autism Dev Disord.

[31] Reynhout G, Carter M (2006). Social Stories™ for children with disabilities. J Autism Dev Disord.

[32] Marshall D, Wright B, Allgar V, Adamson J, Williams C, Ainsworth A (2016). Social Stories™ in mainstream schools for children with autism spectrum disorder: a feasibility randomised controlled trial. BMJ Open.

[33] Williams C, Wright B (2017). A guide to writing social stories™ step-by-step guidelines for parents and professionals.

[34] Constantino JN, Gruber CP (2012). Social responsiveness scale–second edition (SRS-2).

[35] Abidin RR (2012). Parenting stress index.

[36] The EuroQol Group (1990). EuroQol-a new facility for the measurement of health-related quality of life. Health Policy.

[37] Chorpita BF, Yim L, Moffitt C, Umemoto LA, Francis SE (2000). Assessment of symptoms of DSM-IV anxiety and depression in children: a Revised Child Anxiety and Depression Scale. Behav Res Ther.

[38] Law D, Jacob J (2015). Goals and goal based outcomes (GBOs): some useful information.

[39] Pillay M, Alderson-Day B, Wright B, Willaims C, Urwin B (2011). Autism Spectrum Conditions--Enhancing Nurture and Development (ASCEND): An evaluation of intervention support groups for parents. Clin Child Psychol Psychiatry.

[40] Schulz KF, Altman DG, Moher D (2010). CONSORT 2010 statement: updated guidelines for reporting parallel group randomised trials. BMC Med.

[41] Strauss A, Corbin J (1998). Basics of qualitative research techniques.

